# The role of physical literacy and mindfulness on health-related quality of life among college students during the COVID-19 pandemic

**DOI:** 10.1038/s41598-023-50958-9

**Published:** 2024-01-02

**Authors:** Tian Yu Gao, Fu Hua Huang, Ting Liu, Raymond Kim Wai Sum, Jin De Liu, Di Tang, Ding Yi Cai, Zi Kang Jiang, Rui Si Ma

**Affiliations:** 1 School of Physical Education, Jinan University, Guangzhou, Guangdong China; 2 Faculty of Medicine, Macau University of Science and Technology, Taipa, Macau China; 3 School of Nursing, Sun Yat-sen University, Guangzhou, China; 4 Department of Sports Science and Physical Education, Faculty of Education, The Chinese University of Hong Kong, Shatin, Hong Kong China

**Keywords:** Human behaviour, Quality of life

## Abstract

This study aimed to examine the role played by the physical literacy and mindfulness in the health-related quality of life (QoL) of college students. In early 2022, 24,236 college students from three universities in southern China were recruited in the study. R software and the lavvan package was utilized to build the structural equation model. The measurement model was composed of three latent factors (physical literacy, mindfulness, and quality of life) and 16 observed variables in total. The results of the measurement model indicated goodness fit with *p* > .05 in Chi-square result, and GFI = .92. In addition, the comparative fit index (.92), Tucker–Lewis index (.91), root-mean-square error of approximation (.07), and root of mean square residual (.11) were in accord with the cutoff model-fit criteria. The results confirm that physical literacy and mindfulness can play a significant and positive role in the structural equation model of quality of life. In addition, this study provides initial evidence that mindfulness and physical literacy could potentially buffer declines in student QoL during the COVID-19 pandemic. Moreover, this study is the first to develop a structural equation model of QoL with multiple indicators, making it a strong addition to existing research on QoL during a pandemic.

## Introduction

The coronavirus disease 2019 (COVID-19) pandemic has turned life upside down for college students worldwide. As classes moved online and campuses shut down, students have been isolated from friends, professors, and support systems. Quarantines, lockdowns, and fears of the virus have led to increased anxiety and depression^1,2^. At a time of life meant for growth and exploration, many students feel their quality of life (QoL) rapidly declining^3^. Understanding and addressing declines in QoL among college students during the COVID-19 pandemic or any similar future pandemic diseases is a significant problem. QoL is defined as an individual’s perception of his/her position in life in the culture and value system context where he/she belongs, which also involves his/her goals, perspectives, standards, and concerns^4^. QoL is a broad, multidimensional, and polysemic concept influenced in complex ways by a person’s physical health, mental state, degree of independence, social relationships, and relationship to salient features of one’s environment.

Previous studies have shown the profound impacts pandemics can have on mental health and QoL^5–7^. The COVID-19 pandemic specifically has been linked to increased anxiety, depression, stress, and loneliness among college students across the globe^8–12^. Assessing and promoting the health-related QoL of college students during this crisis is vital to supporting their physical and mental wellbeing^5^.

College students are at an uncomfortable stage in their lives, facing family expectations, the pursuit of personal achievement, and the trade-off among study, work, and life^6^. Thus, college students tend to get lost under pressure, leading to the development or exacerbation of certain psychological disorders and unhealthy lifestyles^7,8^, particularly during the COVID-19 pandemic^3^. Both the physical and mental health-related QoL of college students have been affected^3^. Understanding and addressing this decline in QoL is critically important as students face immense challenges to their wellbeing during the pandemic.

The QoL of college students is the unity of physical and mental^9^. Similarly, any experiment attempting to improve QoL must also consider the unity of the physical and mental dimensions simultaneously. This idea is very similar to another concept, also proposed when the world faced a serious lack of physical and physical literacy. The objective is to encourage people to take responsibility for their bodies and minds by re-establishing the core idea of the monism of mind and body^10^. Physical literacy is a multidimensional concept, and different cultures worldwide have developed different ways of interpreting it. The first systematic review of physical literacy summarizes and clarifies its attributes, philosophical underpinnings, and theoretical foundations^11^. Scholars and practitioners have been very careful to map out in words the paths of physical literacy development in their respective cultures. According to the model proposed by Whitehead, physical literacy develops from three domains: affective, physical, and cognitive. At the macro level, physical literacy emphasizes the inseparability of body and mind, with several dimensions interacting with one another. At the micro level, physical literacy emphasizes lifelong movement and positive attitudes^10,12^. Moreover, studies suggested that there may be a factor of resistance in the middle of physical literacy and mental health^12^. Within the overall concept of cognitive, behavioral, and effective health, physical literacy has great potential for improving mental health^13,14^. This characteristic should be taken more seriously under adversity, such as the COVID-19 pandemic.

Stress is prevalent in modern society and has become a significant global health problem. Unfortunately, the COVID-19 crisis has exacerbated this trend^15^. Evidence shows that depression symptoms increased in 14.6–48.3% of the general population in several countries worldwide during the pandemic^16^. Research suggested that high stress levels can negatively affect physical and mental health, thereby decreasing QoL. At the college level, student stress overload is more common. Many studies used mindfulness training techniques to alleviate stress and reduce symptoms of depression and anxiety^17^. Mindfulness is often described as “the development of awareness through purposeful, present, non-judgmental attention to moment-to-moment experience”^18^. Mindfulness is moment-to-moment awareness cultivated through purposeful attention to the experience of the present moment in a non-judgmental manner^17^. During the COVID-19 pandemic, people face unknown risks and thus increase psychological stress caused by fear, and this protective mechanism keeps people’s mental health from wide fluctuations leading to mental illness^19,20^. Given the ubiquitous stressors of college life—now exacerbated by the uncertainty and grief brought on by the COVID-19 crisis—fostering protective factors in college students may be a more achievable path to positive mental health^21^.

While extensive research has examined QoL and associated factors in university students, few studies have focused specifically on the impacts of the COVID-19 pandemic on this population. A significant gap exists in understanding how pandemic-related lifestyle changes and distress may be affecting QoL across physical, psychological, social and environmental domains^22^. Furthermore, despite growing evidence on the benefits of mindfulness and physical literacy for well-being, no studies have investigated their potential to mitigate declining QoL in students during the pandemic. This study seeks to address these critical gaps by assessing overall QoL, and its relationship between physical literacy and mindfulness in Chinese university students during COVID-19. Examining these underexplored associations will provide novel and timely insights into evidence-based pathways for promoting student resilience and protecting QoL during the ongoing pandemic. Findings will also inform interventions to safeguard student well-being in future crises.While the scope is limited to Chinese universities, this demographic represents a large population experiencing significant pandemic-related QoL impacts.

This study is underpinned by a biopsychosocial framework for understanding health and wellbeing. Engel's biopsychosocial model posits that biological, psychological, and social factors interact to shape human health outcomes^23^. This aligns with the multidimensional nature of QoL, which encompasses physical, mental, and social dimensions. The concepts of mindfulness and physical literacy also fit within this framework. Research shows mindfulness can improve cognitive flexibility, emotion regulation, and resilience to stress^24^. Physical literacy emphasizes embodied knowledge, physical confidence, and integration of mind–body experiences^25^. Higher physical literacy is associated with greater psychological benefits of physical activity and sport^11^. This study hypothesizes that cultivating mindfulness and physical literacy may help buffer the impacts of COVID-19 related distress on QoL by enhancing psychological flexibility. The biopsychosocial model provides a conceptual basis for examining how these psychological and embodied factors can moderate health-related QoL outcomes. Specifically, the hypotheses of this study are as follows:

*Hypothesis 1: Physical literacy has a direct impact on health-related QoL.*

*Hypothesis 2: Mindfulness has a direct impact on health-related QoL.*

This report is organized into five sections. The introduction establishes the background and motivation for examining QoL in university students during COVID-19. The literature review synthesizes relevant research on QoL, physical literacy and mindfulness. The methods section details the study design, participants, measures, procedures for data collection and analysis. Results are presented for each research objective, summarizing key statistical analyses. The discussion interprets the findings, notes limitations, and outlines implications for promoting QoL among college students during the pandemic.

## Methods

### Design and participants

Cross-sectional data were obtained from a 4-year longitudinal study that monitored changes in health-related factors throughout an undergraduate's life under natural conditions. The study followed the physical and mental health of college students at three colleges in southern China for four years beginning in 2022. The data used in this study came from the 2022 baseline. The study was a collaborative effort between South China University of Technology, South China Normal University, and Jinan University. Questionnaires were disseminated via an online platform. A total of 25,391 undergraduates took part in the study, with 24,236 of them completing the questionnaires. The response rate was 95.45%.

### Ethical approval and informed consent

In this study, we adhered to ethical principles and obtained ethical approval from the Institutional Review Board (IRB) of Jinan University (Approval No. JNUKY-2021-008). Prior to obtaining ethical approval, we submitted a detailed research plan and an ethics application, including information on the research objectives, methods, risk assessment, and protective measures. The research protocol was reviewed by the ethics committee, which confirmed its compliance with ethical guidelines and regulatory requirements.

Before the commencement of the study, we provided detailed research information to all participants and addressed any questions they may have had. Informed consent was collected using a consent form, which included information on the purpose of the study, procedures involved, potential risks and benefits, and the participants' voluntary right to withdraw from the study. We emphasized the participants' freedom to choose whether or not to participate and ensured the confidentiality of their personal information.

### Sample size

The study utilized a large sample of 24,236 undergraduate students from three universities in China. According to Wolf et al.^26^, a sample size of over 200 is considered adequate for SEM analysis when models include around seven latent variables, as in the present study. Furthermore, SEM could detect a small standardized path coefficient of 0.10 with 80% power and an alpha level of 0.05. The large sample size provided high statistical power to detect small effects and ensured stable estimates of parameters in the complex hypothesized model^27^. In the current sample of over 24,000 students, there was sufficient power to detect very small effects. This allowed testing the hypothesized relationships between QoL, physical literacy, and mindfulness. The robust sample also reduced issues such as non-convergence that can affect SEM solutions with small samples^28^. Overall, the substantial sample size was a strength of the study, enhancing statistical power and ensuring the complex model could be estimated accurately and reliably.

### Outlier handling

Prior to analysis, all variables were examined for outliers. Box plots identified extreme values outside 3 standard deviations for each variable. To minimize influence of outliers, Winsorization was applied to replace the most extreme values with adjacent highest or lowest values. This helped obtain more robust statistical results^29^.

### Measures

Health-related QoL was measured through the Mandarin 36-Item Short Form Health Survey (SF-36). It contains 36 items measuring 8 domains—physical functioning (PF), role-physical (RP), bodily pain (BP), and general health (GH). The mental component includes validity (VT), social function (SF), role-emotional (RE), and mental health (MH). Domain scores are calculated by summing items and transforming raw scores to a 0–100 scale, with higher scores indicating better QoL^30^.The Chinese version of the SF-36 has demonstrated good reliability and validity in the Chinese population, with Cronbach's alpha values ranging from 0.72 to 0.88 across domains and 2-week test–retest reliability coefficients range from 0.66 to 0.94^31^.

Physical literacy was assessed through the Perceived Physical Literacy Instrument^32^. Its simplified Chinese version contains 8 items assessing (1) motivation, (2) confidence and physical competence, and (3) interaction with the environment domains on a 5-point Likert scale.. Motivation examines whether individuals will maintain a positive attitude toward physical activity throughout their lives. Confidence and physical competence test whether people can act with confidence and poise in various challenging situations. Then, interaction with the environment assesses whether individuals can interact with their environment in the context of each day^10^. The simplified Chinese version of the Perceived Physical Literacy Instrument has demonstrated adequate reliability (Cronbach’s alpha = 0.86) and validity through confirmatory factor analysis (CFA). The CFA showed good model fit (RMSEA = 0.03; AGFI = 0.96; NFI = 0.97; CFI = 0.99) with factor loadings ranging from 0.60 to 0.92^33^. Mindfulness was assessed using the Five Facet Mindfulness Questionnaire^34^. This 39-item questionnaire measures five facets of mindfulness: (1) observing, (2) describing, (3) acting with awareness, (4) nonjudging, and (5) nonreactivity on a 5-point Likert scale. The observing facet measures the tendency to notice or attend to internal and external experiences. Describing is a measure of the tendency to use language to describe and label these experiences. Acting with awareness refers to giving full awareness and undivided attention to the activity or experience at hand. Nonjudging means standing in a non-evaluation position to inner experience. Finally, nonreactivity is the tendency to allow thoughts and feelings to appear and disappear without being drawn to or carried away by them. Facet scores are computed by summing component items. It has demonstrated good internal consistency with alpha ranged from 0.84 to 0.93^35^. The Chinese FFMQ also has acceptable reliability (alpha = 0.44–0.84) and one month test–retest reliability coefficients range from 0.44 to 0.74^36^.

### Statistical analysis

Structural equation modeling (SEM) was utilized as the primary analysis method due to its advantages in modeling complex relationships and testing mediation effects^37^. In contrast to simpler regression models, SEM allows the modeling of relationships among multiple predictor and criterion variables simultaneously. It accounts for measurement error in observed variables when estimating effects on latent constructs, which provides more accurate estimates of true scores^38^. As this study involves examining the potential mediating effects of mindfulness and physical literacy on the health-related QoL, SEM is ideal for testing the direct and indirect pathways in this multidimensional framework^39^. By incorporating latent variables, SEM also reduces issues related to multicollinearity that can negatively impact mediation analyses in regression^40^. Given the complex interrelationships hypothesized among health-related QoL, mindfulness, and physical literacy, SEM was determined to be the optimal analysis technique.

In this study, R software was utilized for data analysis, and the lavvan package was used to build SEM^41^. Descriptive statistics were employed to delineate the attributes of the study participants. Prior to conducting the analysis, assumptions of normality, linearity, and homoscedasticity were scrutinized and found to be well-founded^42^. Residual scatterplots showed random patterns, confirming assumptions of homoscedasticity, linearity, and uncorrelated errors. Skewness and kurtosis values were also within acceptable ranges. Overall, the data was deemed suitable for SEM analysis^27^. To evaluate the associations among the observed variables, the bivariate linear correlation coefficient was computed^42^. Subsequently, regression analysis was utilized to delve deeper into the relationships among the three latent variables.

### Model identification

In SEM, the total score of physical literacy, mindfulness, and QoL were treated as latent variables. Their respective components, such as confidence and physical competence, acting with awareness, and physical component summary were considered as observed variables. Subsequently, We tested the first hypothesis by analyzing the path relationship between the observed variables *(1) Confidence and physical competence, (2) Motivation, (3) Interaction with the environment*) and QoL. The second hypothesis was tested by analyzing the path relationship between the observed variables *(1) Observing, (2) Describing, (3) Acting with awareness, (4) Nonjudging, (5) Nonreactivity*) and QoL.

Prior to estimating the structural equation model, model identification was verified. The model contained 16 observed variables and 3 latent variables. With 16 observed variables, the model had 136 data points (16*(16 + 1)/2). Since the number of data points exceeded the number of parameters to estimate, the model was overidentified and thus considered identifiable. Additionally, each latent variable had at least two indicators, and the residual variances of observed variables were not constrained to zero. These criteria further ensured proper identification of the measurement and structural models^27^. Hence, the proposed model was confirmed to be statistically identifiable, allowing unique estimates of all model parameters to be obtained based on the empirical data using maximum likelihood estimation. With model identification verified, structural equation modeling proceeded to test the hypothesized relationships between QoL, mindfulness, and physical literacy.

## Results

The study included 24,236 undergraduate students aged 18–22 years (*M* = 19.58, *SD* = 0.42). The sample comprised 11,829 males (48.8%) and 12,407 females (51.2%). Table 1 shows the descriptive statistics of the mean scores and standard deviations (*SD*) of each variable. Cronbach alpha (α) value was above 0.7, which indicates enough internal consistency reliability.

**Table 1:** Descriptive statistics of variables in the study.

Variables	Mean	SD	Cronbach alpha (α)
Physical literacy	31.45	5.25	.87
Confidence and physical competence (PL1)	11.36	2.53	
Motivation (PL2)	12.72	1.88	
Interaction with the environment (PL3)	7.38	1.93	
Mindfulness	124.01	12.81	.83
Observing (M1)	28.65	5.86	
Describing (M2)	26.01	5.29	
Acting with awareness (M3)	25.87	5.87	
Nonjudging (M4)	21.02	5.07	
Nonreactivity (M5)	22.46	4.23	
QoL	77.70	14.90	.80
Physical component summary	82.25	14.89	
Mental component summary	73.16	18.28	

Table 2 shows Pearson correlations among each observed variable. The results indicated that most variables were correlated with one another.

**Table 2:** Pearson correlations among observed variables of physical literacy, mindfulness, and QoL.

	1	2	3	4	5	6	7	8	9	10
1 Confidence and physical competence (PL1)	–									
2 Motivation (PL2)	.70^a^	–								
3 Interaction with the environment (PL3)	.68^a^	.68^a^	–							
4 Observing (M1)	.26^a^	.27^a^	.40^a^	–						
5 Describing (M2)	.26^a^	.23^a^	.36^a^	.39^a^	–					
6 Acting with awareness (M3)	.10	.02	.02	.21^a^	.34^a^	–				
7 Nonjudging (M4)	.10	.09	.20^a^	.54^a^	.01	.51^a^	–			
8 Nonreactivity (M5)	.25^a^	.31^a^	.36^a^	.60^a^	.20^a^	.38^a^	.56^a^	–		
9 Physical component summary	.38^a^	.39^a^	.26^a^	.07	.17^a^	.30^a^	.11	.01	–	
10 Mental component summary	.29^a^	.23^a^	.26^a^	.18^a^	.36^a^	.40^a^	.12	.16^a^	.61^a^	–

^a^Correlation is significant at the .01 level (two-tailed).

The measurement model was composed of three latent factors (i.e., physical literacy, mindfulness, and QoL) and 16 observed variables in total. The results of the measurement model indicated goodness fit with *p* > .05 in Chi-square result, and GFI = .92. In addition, the comparative fit index (.92), Tucker–Lewis index (.91), root-mean-square error of approximation (.07), and root of mean square residual (.11) were in accord with the cutoff model-fit criteria^43,44^. Table 3 presents the standardized coefficients (*β*) for each overserved variable with loadings ranging from .46 to .91, excluding the lowest .08. In summary, the measurement model in this study has a reasonable factor structure and sufficient convergent VT.

**Table 3:** Results for the measurement model.

Latent construct	Observed variables	Estimate	SE	*β*
Physical literacy	Confidence and physical competence (PL1)	1.00	–	.72^a^
	Motivation (PL2)	.73	.07	.71^a^
	Interaction with the environment (PL3)	.78	.07	.74^a^
Mindfulness	Observing (M1)	1.00	–	.86^a^
	Describing (M2)	.55	.06	.53^a^
	Acting with awareness (M3)	.09	.09	.08
	Nonjudging (M4)	.43	.06	.43^a^
	Nonreactivity (M5)	.58	.05	.69^a^
QoL	Physical component summary (PCS)	1.00	–	.91^a^
	Mental component summary (MCS)	2.26	.39	.91^a^
PCS	PF (PC1)	1.00	–	.46^a^
	RP (PC2)	2.34	.41	.48^a^
	BP (PC3)	1.78	.28	.62^a^
	GH (PC4)	2.40	.34	.78^a^
MCS	VT (MC1)	1.00	–	.87^a^
	SF (MC2)	1.16	.10	.66^a^
	RE (MC3)	1.26	.16	.48^a^
	MH (MC4)	.94	.06	.81^a^

^a^*p* < .001.

**Figure 1:**
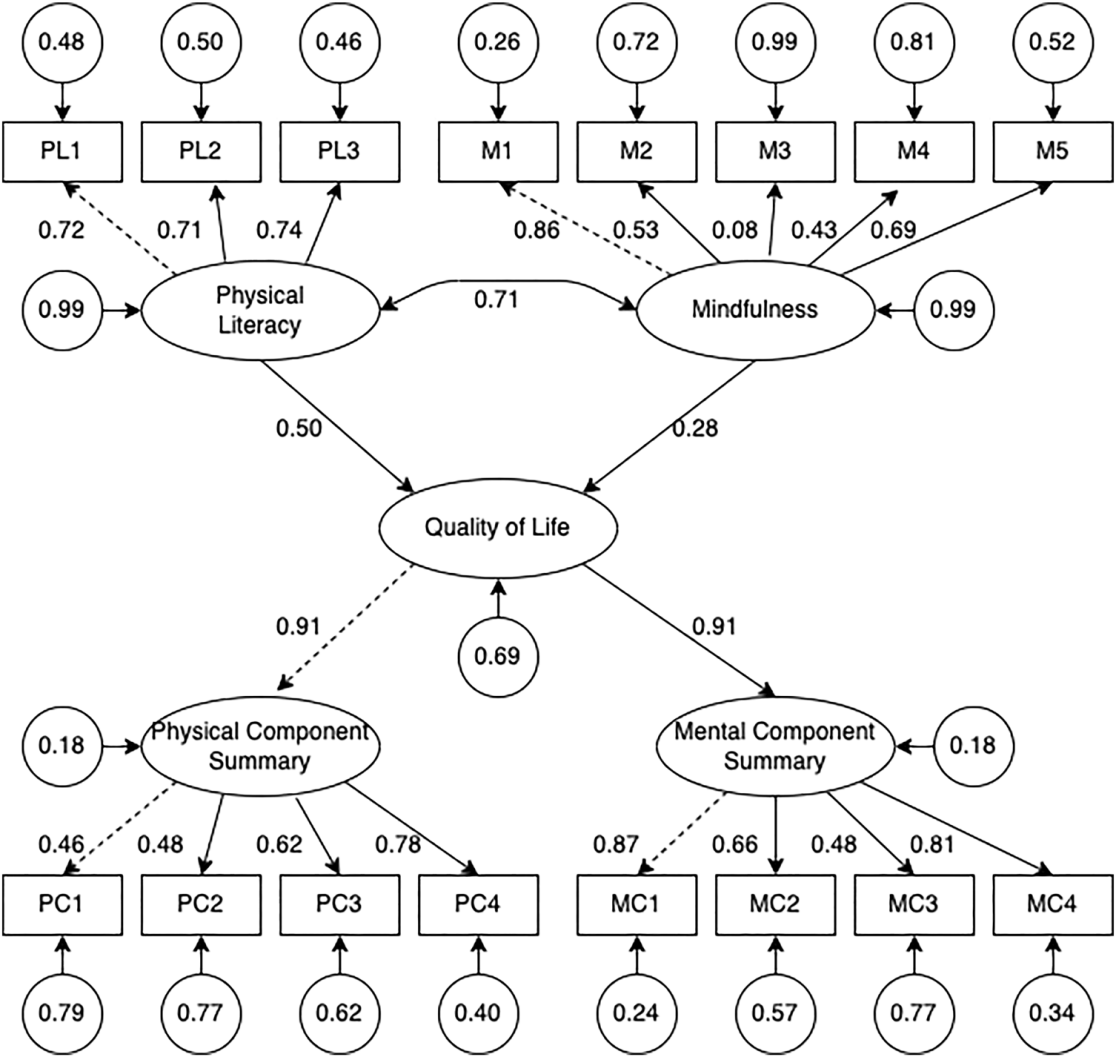
The path relationships in Quality of Life examined using structural equation model.

Based on the previous hypotheses and Pearson correlation results, we conducted SEM with physical literacy and mindfulness as predictors and QoL as the outcome variable, which includes physical and mental elements (Fig. 1).

## Discussion

This study explored the role of physical literacy and mindfulness on QoL. QoL is divided into physical and mental elements, which are equally important in contributing to the total score. Health-related QoL has been the subject of international attention. The term refers to a comprehensive subjective model of health that covers physical, social, psychological, and functional aspects of an individual’s well-being and is a multidimensional subjective construct^45,46^. The significance of investigating this concept is to guide the organization of resources and decision-making processes to promote the QoL of younger adults^47,48^. Although QoL is so critical and should be appreciated, the QoL of college students continues to decline. Hence, to address this situation, this study attempted to develop a structural equation model using physical literacy and mindfulness, hoping that this model may provide a new perspective for future intervention design.

### The effect of physical literacy on health-related QoL

As expected, this study found that physical literacy positively predicted QoL. This finding implies that individuals with stronger physical literacy could enjoy a healthier life. In particular, physical activity can improve well-being and overall health^49^. Physical literacy builds on physical activity with a greater emphasis on taking responsibility for one’s body and the need to maintain it over time^50^. Physical literacy and physical activity are inherently interconnected^51^, and this relationship allows physical literacy to be effective in improving overall QoL.

As the primary observed variable defined in the construct of QoL, physical literacy encompasses more than just physical competence; it can also help promote mental health and support young people in their pursuit of a harmonious state of physical and mental health^12^. Regarding specific dimensions, the SEM results suggested that motivation (*β* = 0.71, *p* < 0.01) and environment interaction (*β* = 0.74, *p* < 0.001) were directly and positively associated with QoL.. We can observe that confidence and physical competence present an indirect effect on the model of physical literacy, which was considered the most dominant influence in previous studies^52^. By contrast, in SEM, owing to the presence of mindfulness and other well-being-related variables, the dimension of confidence and physical competence needs to be linked to physical literacy by other mediated means. Another possible explanation is that the data were collected during the COVID-19 pandemic, when the students’ physical competence and self-confidence needed to be activated by other variables under special periods^53^. From another aspect, motivation and interaction with the environment fit well in the model. The significance of motivation for improving QoL needs to be considered in the context of the overall concept of physical literacy, which promotes a responsible attitude toward the body and a constant desire to exercise^54^. This case was particularly notable during the COVID-19 pandemic when people passionate about physical activity sought opportunities to improve their QoL by engaging in physical activity at home or in isolated locations, even in the face of isolation. Finally, the observed variable of interaction with the environment contributed the most relevance to the model. Effective interaction with the environment stems from the attributes of embodied view in physical literacy. Effective social and communication skills will adequately avoid isolation in the COVID-19 pandemic and maximize one’s well-being and overall QoL, given limited resources.

Overall, these findings emphasize that physical literacy goes beyond physical competence to promote holistic wellbeing. Fostering motivation and adaptability are key to sustaining an active lifestyle during challenging circumstances like COVID-19. The dimensions of physical literacy are interconnected and can compensate when particular aspects like competence are disrupted. These results demonstrate the multidimensional mechanisms through which physical literacy enhances health-related QoL.

### The effect of mindfulness on health-related QoL

Standardized mindfulness-based interventions combine the essence of traditional mindfulness practice with contemporary psychological practice. Moreover, such interventions have been shown to be effective in improving a range of clinical and non-clinical cardiac outcomes, such as anxiety, current depressive symptoms, stress, and chronic pain, and can reduce the risk of recurrence of depression^55–58^. Mindfulness has been proven to significantly improve QoL^59,60^. However, previous studies made the link to QoL in the form of cognition, making a link to the psychological element of QoL through the pathway of mental health and well-being. The model in this study considers the psychological element of QoL while adding physical literacy, which also considers the physical element as a latent variable. The results of the test show that mindfulness also has a significant correlation with QoL.

According to the SEM model results, no direct link exists between observing and mindfulness, and other variables influence them. This result is consistent with previous research findings that observing requires a certain amount of meditative experience to be effective^34^. In an experiment using mindfulness to intervene in mental health, the observing facet needed to be combined with other psychological adjustment variables to have a significant intervention effect in a group with no meditation experience^36^. In addition, the absence of meditation experience can limit participants’ ability to perceive internal and external stimuli. The facet of describing (β = 0.53, *p* < 0.001), nonjudging (β = 0.43, *p* < 0.001), and nonreactivity (β = 0.69, *p* < 0.01) showed a strong positive relationship with overall mindfulness, namely, verbally expressing inner experience, perceptions, and emotions clearly and maintaining a neutral attitude toward them without judging or reacting. This state of neutrality toward one’s inner experience has a significant gain in QoL under stress. Particularly during events such as the COVID-19 pandemic, college students need to deal with great psychological stress, not blindly feel frustrated, understand their own feelings, and not let any negative effects erode their mental health to effectively cope with difficult times and ensure their QoL. The only observed variable that was not significant in the model was acting with awareness. The reason may be that during the epidemic, the isolation caused impairment in the ability of college students to focus on action. Although focusing on current actions did not contribute much to the overall model, its presence as one of the dimensions of positive thinking still supported the other observed variables.

The facets of mindfulness interacted in compensatory ways. For instance, the inability to focus on the present moment (acting with awareness) during the pandemic may have been counterbalanced by describing emotions and practicing nonreactivity. This suggests certain mindfulness skills were more essential for wellbeing in the crisis context. Additionally, among the physical elements under QoL, physical functioning presents a non-direct relationship to body-related QoL, indicating that good or bad physical functioning needs to be combined with other factors, such as mental health, to have an impact on overall QoL. Similarly, the mental element, vitality, must be combined with physical factors to better function in the QoL model.

Overall, these results demonstrate how specific mindfulness facets contribute to this buffering effect on health-related QoL, highlighting important skills like verbal expression, neutrality and nonreactivity. The findings provide insights into how cultivating multidimensional mindfulness can promote wellbeing in students facing pandemic-related adversity.

## Limitations

This study has some key delimitations related to the scope and sample. First, it utilizes a cross-sectional survey design, which provides insights into relationships at one point in time rather than changes over time. The data come from a larger longitudinal study, but this analysis employs the baseline wave only. Second, the sample is delimited to undergraduate students at three universities in southern China. While the large sample enhances generalizability to university students in this region, it does not represent all of China or other countries. Third, a convenience sampling method was used to recruit participants, which could introduce sampling bias compared to random selection. Fourth, the surveys are self-report in nature, relying on students to accurately report their perceptions and behaviors. Future analyses could explore how QoL, mindfulness, and physical literacy change over time. In particular, longitudinal SEM could examine whether changes in mindfulness and physical literacy predict subsequent changes in quality of life. This would provide stronger evidence for the hypothesized causal pathways. Additionally, intervention studies experimentally manipulating mindfulness and physical literacy are warranted. Randomized controlled trials could directly test if increasing mindfulness and physical literacy skills causes improvements in university students' QoL during crises like the COVID-19 pandemic. Comparing students randomly assigned to mindfulness or physical literacy training versus control groups would clarify the causal nature of these relationships.

## Conclusion

This study explores the role of physical literacy and mindfulness on QoL among Chinese college students. QoL consists of equally important physical and mental components that contribute to overall well-being^46^. Understanding factors influencing QoL is critical for promoting the health and wellness of younger adults^48^. However, the QoL of college students has declined, especially during the COVID-19 pandemic^22^. This underscores the need to examine relationships between QoL, mindfulness, and physical literacy among this population.

The findings of this study emphasize the importance of physical literacy and mindfulness for buffering declines in college students' QoL. Physical competence, motivation for physical activity, and environmental interactions were strongly associated with higher QoL. This aligns with research showing physical activity benefits overall health and well-being^49^. Fostering physical literacy encourages lifelong responsible engagement in physical activity^50^, which can promote QoL even during pandemic restrictions. Mindfulness facets such as nonjudgement, nonreactivity, and describing inner experiences also exhibited significant positive relationships with QoL. These skills allow students to cope with psychological distress in adaptive ways to maintain wellness^24^.

This study makes key contributions to understanding factors influencing college student QoL during COVID-19. The results demonstrate how cultivating mindfulness and physical literacy could buffer declines in QoL resulting from pandemic-related mental health issues. These findings have important practical implications for colleges seeking to protect student health and wellness during times of crisis. Mindfulness interventions and physical programs focused on building skills and motivation could be critical supports for maintaining QoL. As the world faces ongoing impacts of COVID-19 and future potential pandemics, promoting mindfulness and physical literacy among students will be key to fostering resilience and well-being.

Overall, this research highlights important pathways for strengthening college students’ QoL during pandemics and beyond. Additional studies are warranted to develop targeted interventions leveraging mindfulness and physical literacy. Helping students build skills to support mental health through mind–body approaches will prepare them to thrive in the face of ongoing life stressors and challenges.

## Data Availability

The datasets used during the current study are available from the corresponding author on reasonable request.

## References

[1] Son C, Hegde S, Smith A, Wang X, Sasangohar F (2020). Effects of COVID-19 on college students’ mental health in the United States: Interview survey study. J. Med. Internet Res..

[2] Kecojevic A, Basch CH, Sullivan M, Davi NK (2020). The impact of the COVID-19 epidemic on mental health of undergraduate students in New Jersey, cross-sectional study. PLoS One.

[3] Leong Bin Abdullah MFI, Mansor NS, Mohamad MA, Teoh SH (2021). Quality of life and associated factors among university students during the COVID-19 pandemic: A cross-sectional study. BMJ Open.

[4] WHOQOL Group (1995). The World Health Organization quality of life assessment (WHOQOL): Position paper from the World Health Organization. Soc. Sci. Med..

[5] Ribeiro ÍJS (2018). Stress and quality of life among university students: A systematic literature review. Health Prof. Educ..

[6] Pedrelli P, Nyer M, Yeung A, Zulauf C, Wilens T (2015). College students: Mental health problems and treatment considerations. Acad. Psychiatry.

[7] Hyun J, Quinn B, Madon T, Lustig S (2007). Mental health need, awareness, and use of counseling services among international graduate students. J. Am. Coll. Heal..

[8] Sharp J, Theiler S (2018). A review of psychological distress among university students: Pervasiveness, implications and potential points of intervention. Int. J. Adv. Couns..

[9] Theofilou P (2013). Quality of life: Definition and measurement. Eur. J. Psychol..

[10] Whitehead M (2010). Physical Literacy: Throughout the Lifecourse.

[11] Edwards LC (2018). ‘Measuring’ physical literacy and related constructs: A systematic review of empirical findings. Sports Med..

[12] Ma R (2021). Relationship among physical literacy, mental health, and resilience in college students. Front. Psychiatry.

[13] Espie CA (2019). Effect of digital cognitive behavioral therapy for insomnia on health, psychological well-being, and sleep-related quality of life: A randomized clinical trial. JAMA Psychiatry.

[14] Almond L (2013). Physical literacy and fundamental movement skills: an introductory critique. ICSSPE Bull. J. Sports Sci. Phys. Educ..

[15] Vindegaard N, Benros ME (2020). COVID-19 pandemic and mental health consequences: Systematic review of the current evidence. Brain. Behav. Immun..

[16] Xiong J (2020). Impact of COVID-19 pandemic on mental health in the general population: A systematic review. J. Affect. Disord..

[17] Khoury B, Sharma M, Rush SE, Fournier C (2015). Mindfulness-based stress reduction for healthy individuals: A meta-analysis. J. Psychosom. Res..

[18] Kabat-Zinn J (2003). Mindfulness-based interventions in context: Past, present, and future. Clin. Psychol. Sci. Pract..

[19] Dawson DL, Golijani-Moghaddam N (2020). COVID-19: Psychological flexibility, coping, mental health, and wellbeing in the UK during the pandemic. J. Context. Behav. Sci..

[20] Adamson MM (2020). International prevalence and correlates of psychological stress during the global COVID-19 pandemic. Int. J. Environ. Res. Public Health.

[21] Yamaguchi K (2020). Role of focusing on the positive side during COVID-19 outbreak: Mental health perspective from positive psychology. Psychol. Trauma Theory Res. Pract. Policy.

[22] Wang C (2020). A longitudinal study on the mental health of general population during the COVID-19 epidemic in China. Brain. Behav. Immun..

[23] Engel GL (1977). The need for a new medical model: A challenge for biomedicine. Science.

[24] Guendelman S, Medeiros S, Rampes H (2017). Mindfulness and emotion regulation: Insights from neurobiological, psychological, and clinical studies. Front. Psychol..

[25] Whitehead M (2001). The concept of physical literacy. Eur. J. Phys. Educ..

[26] Wolf EJ, Harrington KM, Clark SL, Miller MW (2013). Sample size requirements for structural equation models. Educ. Psychol. Meas..

[27] Kline RB (2023). Principles and Practice of Structural Equation Modeling.

[28] Barrett P (2007). Structural equation modelling: Adjudging model fit. Pers. Individ. Differ..

[29] Ghosh D, Vogt A (2012). Outliers: An Evaluation of Methodologies.

[30] Ware JE, Sherbourne CD (1992). The MOS 36-Item Short-Form Health Survey (SF-36): I. Conceptual framework and item selection. Med. Care.

[31] Li L (2003). Chinese SF-36 Health Survey: Translation, cultural adaptation, validation, and normalisation. J. Epidemiol. Community Heal..

[32] Sum RKW (2016). Construction and validation of a perceived physical literacy instrument for physical education teachers. PLoS One.

[33] Ma RS, Sum RKW, Hu YN, Gao TY (2020). Assessing factor structure of the simplified Chinese version of Perceived Physical Literacy Instrument for undergraduates in Mainland China. J. Exerc. Sci. Fit..

[34] Baer RA, Smith GT, Hopkins J, Krietemeyer J, Toney L (2006). Using self-report assessment methods to explore facets of mindfulness. Assessment.

[35] Christopher MS, Neuser NJ, Michael PG, Baitmangalkar A (2012). Exploring the psychometric properties of the five facet mindfulness questionnaire. Mindfulness (N. Y.).

[36] Deng Y-Q, Liu X-H, Rodriguez MA, Xia C-Y (2011). The five facet mindfulness questionnaire: Psychometric properties of the Chinese version. Mindfulness (N. Y.).

[37] Hoyle RH (1995). Structural Equation Modeling: Concepts, Issues, and Applications.

[38] Schumacker RE, Lomax RG (2004). A Beginner’s Guide to Structural Equation Modeling.

[39] Iacobucci D (2008). Mediation Analysis.

[40] Frazier PA, Tix AP, Barron KE (2004). Testing moderator and mediator effects in counseling psychology research. J. Couns. Psychol..

[41] Rosseel Y (2012). lavaan: An R package for structural equation modeling. J. Stat. Softw..

[42] Rodgers JL, Nicewander WA (1988). Thirteen ways to look at the correlation coefficient. Am. Stat..

[43] Hu L, Bentler PM (1999). Cutoff criteria for fit indexes in covariance structure analysis: Conventional criteria versus new alternatives. Struct. Equ. Model. A Multidiscip. J..

[44] Byrne BM (2016). Structural Equation Modeling with AMOS.

[45] Wee CC, Davis RB, Hamel MB (2008). Comparing the SF-12 and SF-36 health status questionnaires in patients with and without obesity. Health Qual. Life Outcomes.

[46] Corica F (2006). Construct validity of the short form-36 health survey and its relationship with BMI in obese outpatients*. Obesity.

[47] Solans M (2008). Health-related quality of life measurement in children and adolescents: A systematic review of generic and disease-specific instruments. Value Health.

[48] Ravens-Sieberer U (2014). Subjective well-being measures for children were developed within the PROMIS project: Presentation of first results. J. Clin. Epidemiol..

[49] World Health Organization. Physical activity. (2022). Available at: https://www.who.int/news-room/fact-sheets/detail/physical-activity.

[50] Whitehead M (2019). Physical Literacy Across the World.

[51] Ma RS, Sum RKW, Li MH, Huang Y, Niu XL (2020). Association between physical literacy and physical activity: A multilevel analysis study among Chinese undergraduates. Int. J. Environ. Res. Public Health.

[52] Choi SM, Sum RKW, Leung EFL, Ng RSK (2018). Relationship between perceived physical literacy and physical activity levels among Hong Kong adolescents. PLoS One.

[53] Jefferies P, Ungar M, Aubertin P, Kriellaars D (2019). Physical literacy and resilience in children and youth. Front. Public Health.

[54] Giblin S, Collins D, Button C (2014). Physical literacy: Importance, assessment and future directions. Sports Med..

[55] Green SM, Bieling PJ (2012). Expanding the scope of mindfulness-based cognitive therapy: Evidence for effectiveness in a heterogeneous psychiatric sample. Cogn. Behav. Pract..

[56] Teasdale JD (2000). Prevention of relapse/recurrence in major depression by mindfulness-based cognitive therapy. J. Consult. Clin. Psychol..

[57] Strauss C, Cavanagh K, Oliver A, Pettman D (2014). Mindfulness-based interventions for people diagnosed with a current episode of an anxiety or depressive disorder: A meta-analysis of randomised controlled trials. PLoS One.

[58] Chiesa A, Serretti A (2009). Mindfulness-based stress reduction for stress management in healthy people: A Review and meta-analysis. J. Altern. Complement. Med..

[59] Godfrin KA, van Heeringen C (2010). The effects of mindfulness-based cognitive therapy on recurrence of depressive episodes, mental health and quality of life: A randomized controlled study. Behav. Res. Ther..

[60] Kuyken W (2008). Mindfulness-based cognitive therapy to prevent relapse in recurrent depression. J. Consult. Clin. Psychol..

